# CdS-Nanowires Flexible Photo-detector with Ag-Nanowires Electrode Based on Non-transfer Process

**DOI:** 10.1038/srep21551

**Published:** 2016-02-22

**Authors:** Yanli Pei, Ruihan Pei, Xiaoci Liang, Yuhao Wang, Ling Liu, Haibiao Chen, Jun Liang

**Affiliations:** 1 State Key Lab of Optoelectronics Materials & Technologies, School of Microelectronics, Sun Yat-Sen University, Guangzhou 510275, P. R. China; 2 School of Physics & Engineering, Sun Yat-Sen University, Guangzhou, 510275, P. R. China; 3 School of Advanced Materials, Shenzhen Graduated School, Peking University, Shenzhen 518055, P. R. China

## Abstract

In this study, UV-visible flexible resistivity-type photo-detectors were demonstrated with CdS-nanowires (NWs) percolation network channel and Ag-NWs percolation network electrode. The devices were fabricated on Mixed Cellulose Esters (MCE) membrane using a lithographic filtration method combined with a facile non-transfer process. The photo-detectors demonstrated strong adhesion, fast response time, fast decay time, and high photo sensitivity. The high performance could be attributed to the high quality single crystalline CdS-NWs, encapsulation of NWs in MCE matrix and excellent interconnection of the NWs. Furthermore, the sensing performance was maintained even the device was bent at an angle of 90°. This research may pave the way for the facile fabrication of flexible photo-detectors with high performances.

In recent years, wearable electronic devices have been growing rapidly, such as smart watches, smart glasses and wearable cameras, due to the significant applications of health monitoring^1^,^2^. Photo-detector is an important part of wearable electronic devices for sensing light in applications such as wearable cameras^3^. However, conventional photo-detectors structured by films of semiconductor and metal are brittle and they often break or form cracks at small strains, such that they cannot be used in the flexible photo-electronics where folding, stretching, twisting, or serious bending are required. Therefore, nanowires (NWs) percolation networks which present good flexibility have been widely investigated^4^,^5^. In addition, as a functional element, NWs demonstrate high sensitivities in nano-scale photon detection sensors due to their large surface-to-volume ratios^6^.

In design of NWs flexible photo-detectors with fast response time, fast reset time, and high sensitivity, excellent interconnections of the NWs are a critical requirement. In addition, it is difficult to achieve a strong adhesion to the substrate while NWs are deposited on plastic substrate without subsequent treatments^7^. Some methods including encapsulation and burying NW networks had been taken to resolve the adhesion issue^8^,^9^,^10^. However, both methods need complicated transfer process after the NWs network is prepared, and cannot achieve good interconnections between the NWs. It is required to develop a simple method to prepare flexible NWs photo-detectors with good adhesion and excellent NWs interconnections.

CdS is a popular and inexpensive photo-conducting material. Because its working spectrum is similar to that of the human eyes, it is currently used to detect the intensity of light and, thereby, to control the shutter speed of cameras. Nowadays, photo-detectors with CdS nanostructures have received extensive attentions due to their higher sensitivity^11^,^12^. Three kinds of construction strategies have been reported, i.e., ohmic contact, schottky contact, and field enhanced transistor. Using the single nanowire or nanobelt, the field transistor type CdS photo-detectors have been demonstrated with very high sensitivity^13^,^14^,^15^. However, these transistor type photo-detectors require complicated fabrication process including fabrications of back gate electrode, gate dielectric, single nanostructure channel and S/D electrodes. It is difficult to bend or stretch. In addition, the single NWs or nanobelts were transferred randomly and led to a low product yields. Schottky contact type photo-detectors need two types of electrodes, one is ohmic contact and another is schottky barrier contact^16^. In comparison, the resistivity type photo-detectors with ohmic contact are the simplest and the most widely used. Besides CdS-NWs being the sensing channel, using Ag-NWs with high conductivity and flexibility as the conducting electrode is also critical for wearable photo-electronics application.

In this work, flexible resistivity-type photo-detectors based on CdS-NWs percolation network channel and Ag-NWs percolation network electrode were fabricated using a facile non-transfer method. Ag-NWs percolation networks have been suggested as high performance flexible and transparent electrodes^17^. Fast response time, fast decay time, and high photo sensitivity were demonstrated. Meanwhile, the sensing performance was maintained even the device was bent at an angle of 90°.

## Results and Discussion

### Single Crystalline CdS-NWs

**Figure 1:**
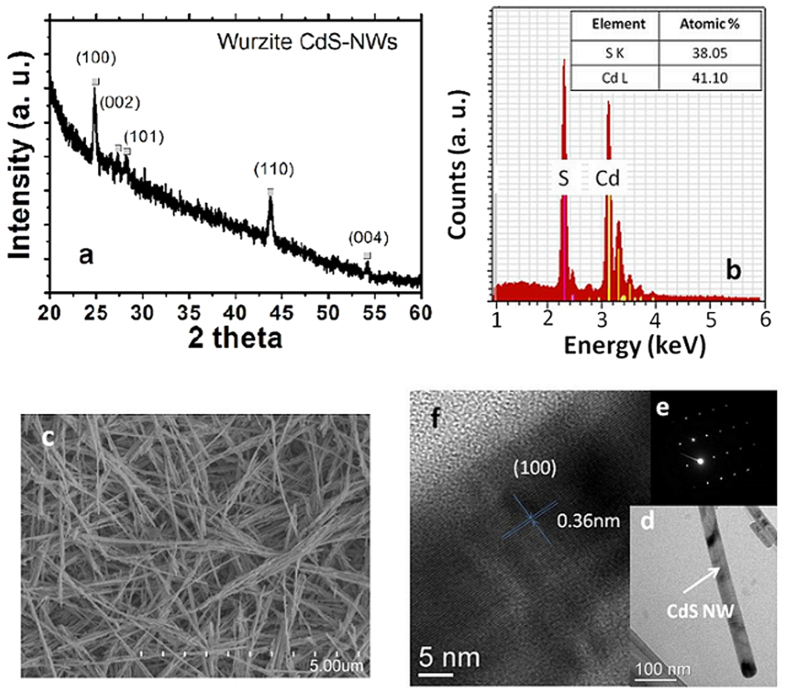
Characterization of CdS-NWs. (**a**) XRD pattern, (**b**) EDX spectrum, (**c**) SEM image, (**d**) Typical TEM image of a CdS-NW, (**e**) SAED pattern corresponded the single CdS-NW, (**f**) HRTEM image of a single CdS-NW with clear lattice fringes.

Figure 1(a) shows a typical X-ray diffraction (XRD) pattern of the as-prepared CdS-NWs powder. All diffraction peaks in the XRD pattern can be indexed to a pure hexagonal wurtzite CdS with lattice constants of a = 4.14 Å and c = 6.72 Å. There results match well with the JCPDS card (Joint Committee on Powder Diffraction Standards, card no. 41-1049). The energy dispersive X-ray spectroscopy (EDX) measurement as shown in Fig. 1(b) indicates that the atom ratio of Cd:S is nearly 1:1. Figure 1(c) is the scanning electron microscopy (SEM) image of CdS-NWs. The length of the CdS-NWs ranges from several to ten micrometers. The microstructure of the CdS-NWs was examined by selected-area electron diffraction (SAED) and high resolution transmission electron microscopy (HRTEM), as shown in Fig. 1(d–f). The CdS-NW is very uniform with a diameter about 35 nm (Fig. 1(d)). The corresponding SAED pattern demonstrates the well single crystallization of the CdS-NW (Fig. 1(e)). The HRTEM image shows clear lattice fringes (Fig. 1(f)). The crystal plane parallel to the NW has a spacing of 3.6 Å, which is consistent with the (100) plane of wurtzite CdS.

### Schematic fabrication of flexible photo-detectors based on CdS-NWs/Ag-NWs

**Figure 2:**
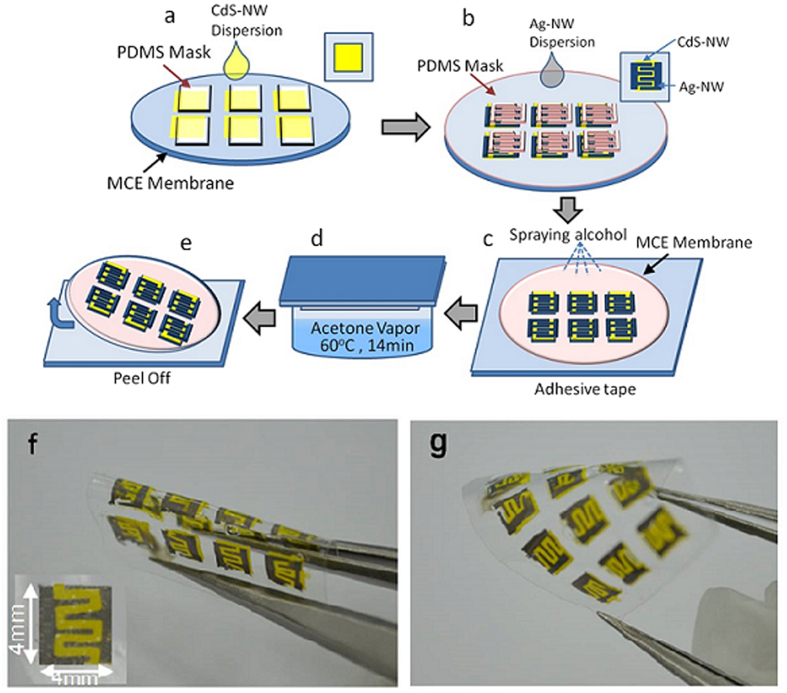
(**a**–**e**) Schematic illustration of the flexible CdS-NWs photo-detector preparation, (**f**,**g**) Digital images of the photo-detector array on the MCE substrate. The inset in (**f**) is an enlarged view of an individual device with Ag-NWs electrodes and CdS-NWs channel.

Illustrations of the lithographic filtration method with non-transfer process are shown in Fig. 2. Different functional components can be integrated into the devices based on this method. Two kinds of filtration masks for CdS-NWs channel and Ag-NWs electrode were prepared using cured polydimethylsiloxane (PDMS) membrane. The soft polymeric masks have similar roles as those hard ultraviolet (UV) masks used in photolithography: to selectively expose areas and obtain desired patterns. The masks are naturally in close contact with the MCE membrane due to the vacuum suction force and the sticky surface of PDMS, which prevents the spreading of the NWs dispersion and ensures well-defined patterns. The CdS-NWs dispersions were filtered to get a uniform CdS-NWs percolation network channel (Fig. 2(a)). The filtration process is fast due to the large pore size (0.45 μm) and high flow rate of the MCE filter membrane. The as-obtained NWs film is quite uniform due to the simultaneous extraction of solvent from the uniformly distributed pores. The thickness of the CdS-NWs film can be controlled by the volume of the NWs dispersion added. Then, the first mask was removed and the second mask for the Ag-NWs fork finger electrode was aligned on the top of the CdS-NWs channel patterns. The Ag-NWs electrode was filtered analogously (Fig. 2(b)). The MCE filter membrane with the patterns of the CdS-NWs channel and the Ag-NWs fork finger electrode was placed on an adhesive tape. After spraying alcohol on the surface of the MCE membrane, the MCE membrane was treated with acetone vapor for 14 min at 60 °C (Fig. 2(c,d)). When the alcohol and acetone vapors were infiltrated into the MCE membrane, the MCE membrane became transparent and pore-free via a re-melting process. Finally, the MCE membrane with photo-detector devices was dried at room temperature and it was peeled off from the adhesive tape. The entire process is very simple and just takes only a dozen minutes. Figure 2(f,g) are the digital images of the flexible photo-detector structures. Because the MCE membrane had experienced the processes from re-melt to solidification, an excellent adhesion of NWs to the MCE substrate was obtained.

### Characterization and evaluation of photo-detectors

**Figure 3:**
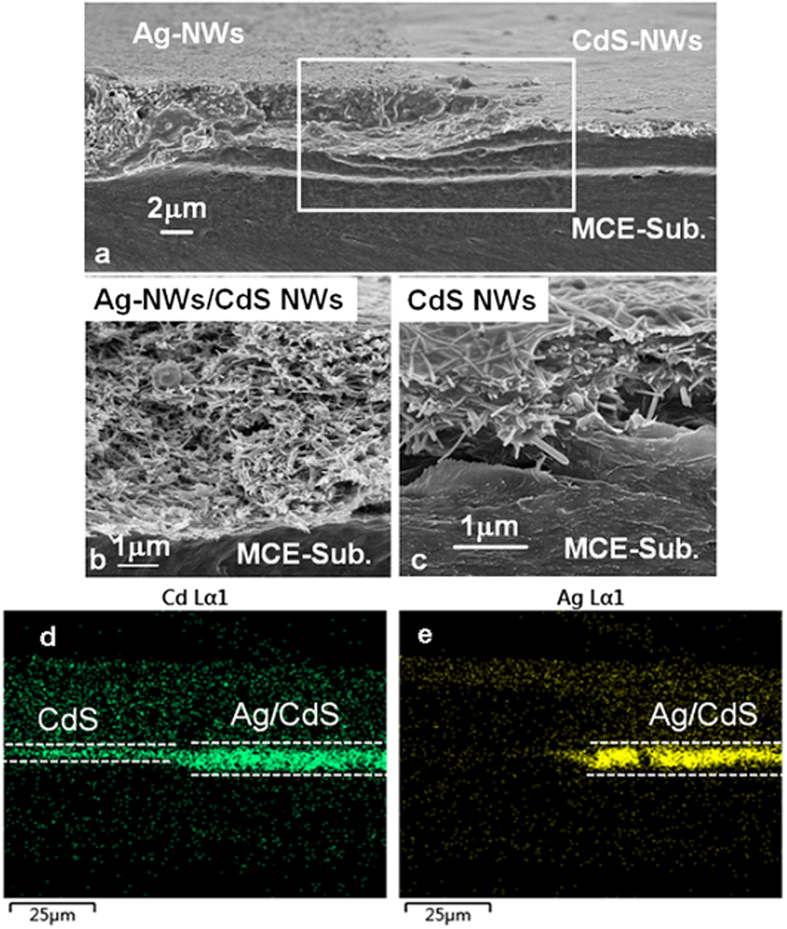
(**a**) SEM cross-sectional view of the Ag-/CdS-NWs overlapping area, (**b**) Enlarged view of the overlapped bi-layer structure, (**c**) Enlarged view of CdS-NWs channel structure, (**d**,**e**) Cd and Ag elements EDX mapping of the square area in (**a**).

Microstructure of the photo-detectors was characterized using SEM combined with EDX elemental mapping. Figure 3(a) shows the cross-sectional view of the Ag-NWs electrode/CdS-NWs channel overlapping area. The enlarged views of the overlapped bilayer structure and the CdS-NWs channel are shown in Fig. 3(b,c), respectively. It is confirmed that the NWs percolation networks are embedded in the MCE matrix. The EDX element mapping of the square area of Fig. 3(a) was carried out to detect the distribution of CdS-NWs and Ag-NWs. As displayed in Fig. 3(d,e), the Ag element just presents in the area of the electrode which is overlapped with Cd element, indicating the infiltration between Ag-NWs and CdS-NWs in the overlapping area. The excellent overlapping should provide good contact of CdS-NWs channel with Ag-NWs electrode. The Cd elements were distributed in the channel area, indicating the CdS-NWs channel was fabricated successfully.

**Figure 4:**
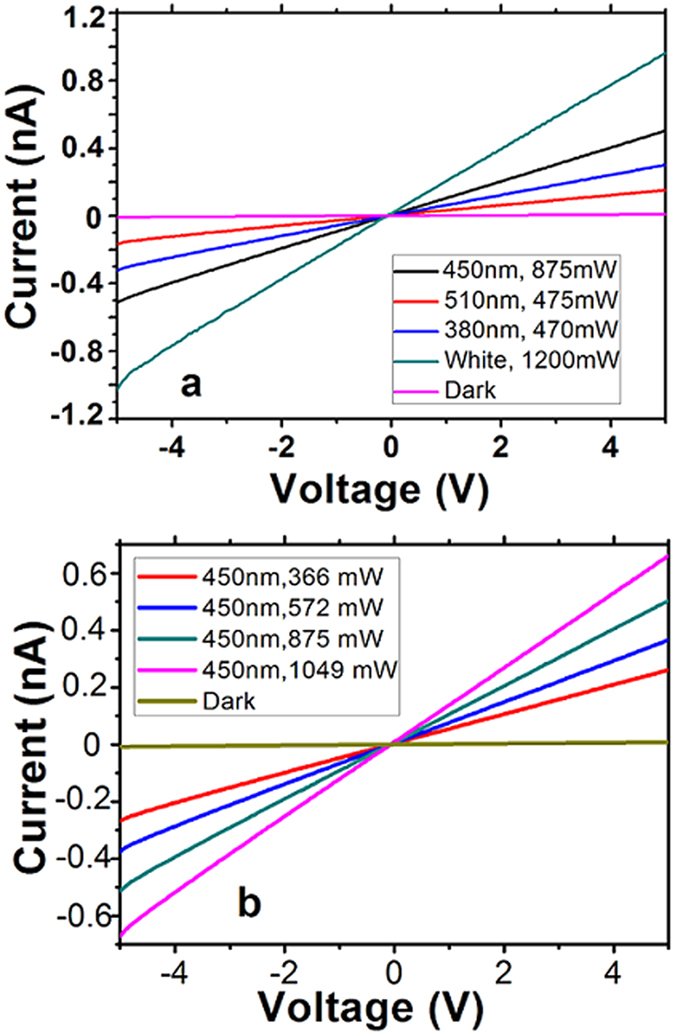
(**a**) I–V curves of the CdS-NWs photo-detector under various wavelength illuminations or dark condition, (**b**) I–V curves of the CdS-NWs photo-detector under blue light illumination (450 nm) with various light powers or dark condition.

Figure 4(a) shows the typical current-voltage (I–V) curves of the MCE-based CdS-NWs photo-detector under a dark condition as well as illuminations with various wavelength lights. The devices were tested without bending. The linear I–V curves indicate the excellent ohmic contact between the electrode and the channel. Under the dark condition, a 3 pA current is recorded at the bias voltage of 2 V. Compared with the dark current, the photocurrent under exposure to light increases drastically. For white light illumination, the ratio of Ion/Ioff is about 120 (light power of 1200 mW), 60 for blue light (450 nm, light power of 875 mW), 27 for green light (510 nm, 475 mW), and 39 for UV light (380 nm, 470 mW), respectively. Figure 4(b) shows the I-V curves under blue light illuminations with various light powers. The photocurrent is increased along with the light power.

**Figure 5:**
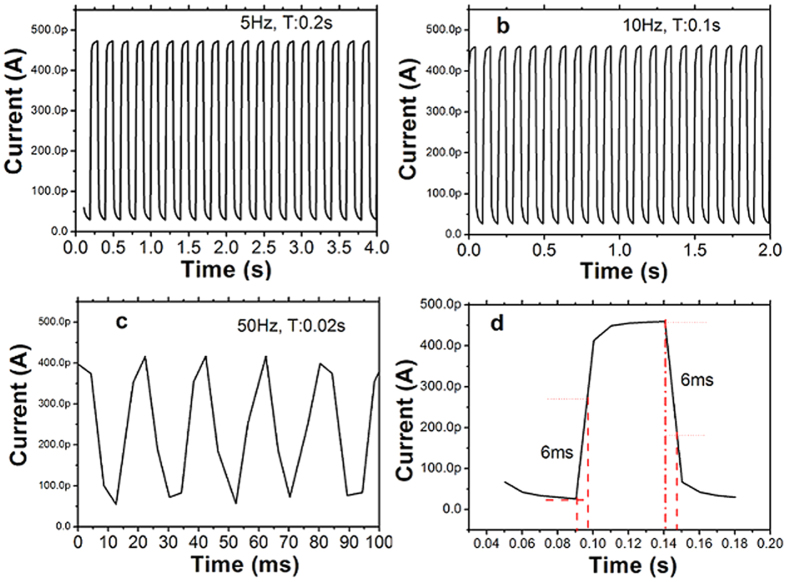
Photo responses of the CdS-NWs photo-detector under blue light illumination (450 nm) with light on/off switching at various frequencies. (**a**) Frequency of 5 Hz, (**b**) Frequency of 10 Hz, (**c**) Frequency of 50 Hz, (**d**) Single modulation cycle of the photo-detector exposed to the blue light (450 nm) with on/off switching frequency of 10 Hz.

The time-dependent photo responses were measured at a constant voltage of 5 V with 450 nm light switching on/off at various frequencies, as shown in Fig. 5(a–c). The photocurrent reaches rapidly to a saturation value under illumination. When the light is turned off, the photocurrent decreases rapidly. The current levels of the on and off states remain nearly constant for ten cycles, indicating the excellent reversibility and stability of this photo-detector. Furthermore, the on/off switching was detected obviously, even at the frequency as high as 50 Hz. The switching speed is one of the most important parameters of photo-detectors. The response time was defined as the time required increasing 63% (1−1/e) from dark current to maximum photocurrent, and the decay time was defined as the time required decreasing to 37% (1/e) of the maximum photocurrent. As shown in Fig. 5(d), both the response time and decay time are 6 ms. In order to compare the photo-response properties of the different NWs photo-detectors, we summarize the crucial parameters in Table 1. As can be seen from Table 1, the CdS-NWs network flexible photo-detector fabricated in this work exhibits a faster photo-response and a relatively high sensitivity.

**Figure 6:**
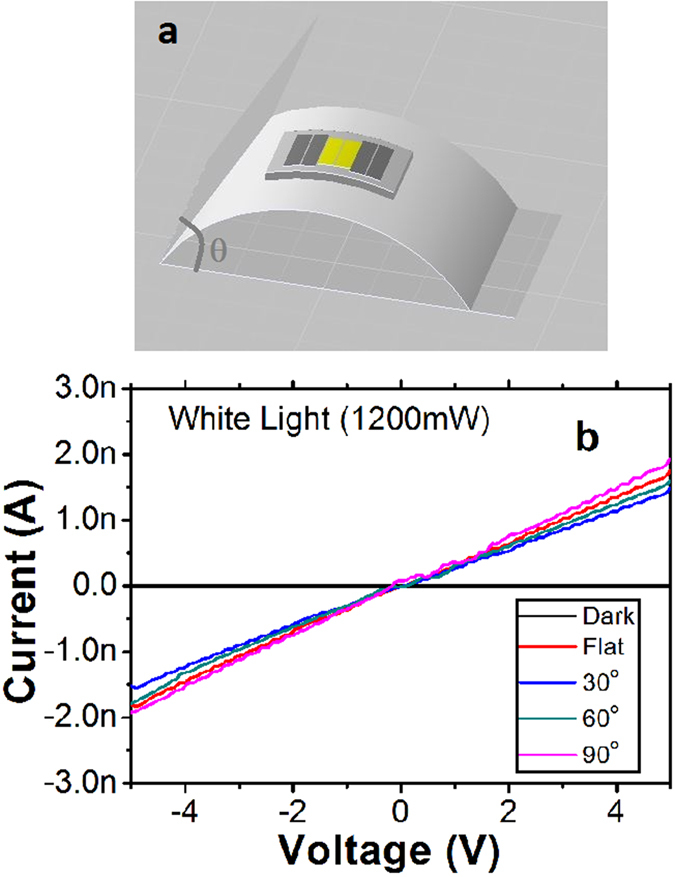
(**a**) Schematic image of the photo-detector bending experience, (**b**) I–V curves of the photo-detector exposed to the white light with various bending angles.

**Figure 7:**
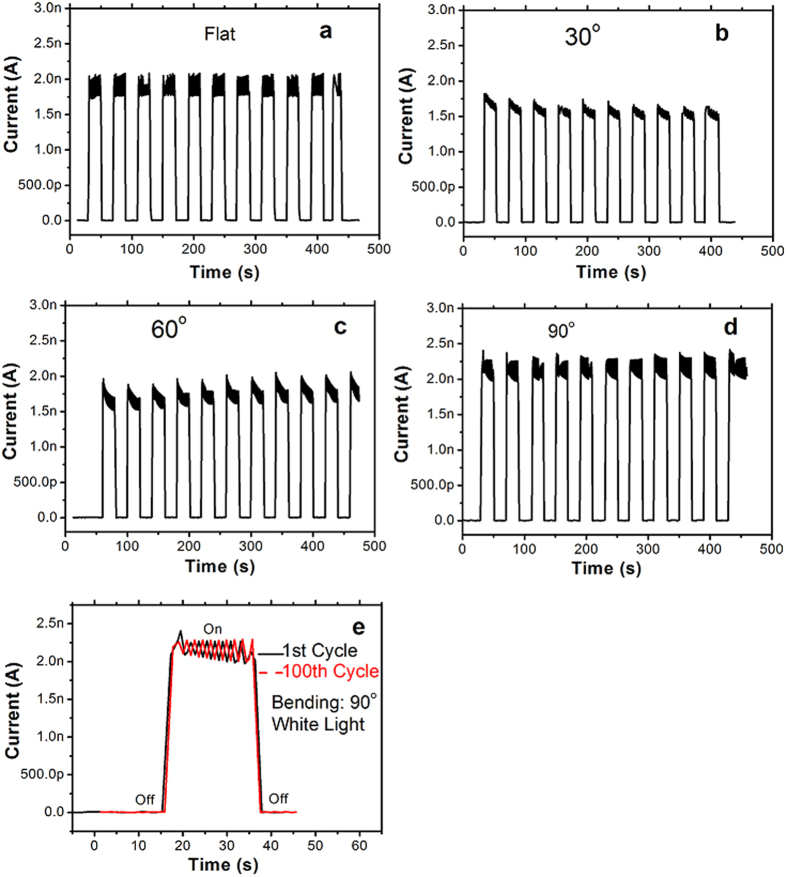
Photo responses of the photo-detector under white light illumination with various bending angles of (**a**) Flat, (**b**) 30°, (**c**) 60°, (**d**) 90°. Cycling stability of the photo-detectors up to 100 cycles at bending angle of 90° (**e**).

The bending experiment was performed by directly bending the devices from flat (0°) to angles of 30, 60, and 90°. Figure 6(a) shows the schematic image of the bending experiment. Figure 6(b) shows the I–V curves of the photo-detector with various bending strain under the white light illumination with the light power of 1200 mW. The photocurrents with good ohmic conducting behaviors are observed under various bending strains. Figure 7(a–d) shows the time-dependent photo response for different bending angles. Figure 7(e) shows the comparison between the properties of the first and 100th cycle at bending angle of 90° under white light illumination. Apparently, there is basically no change in the response. Thus, these results clearly indicate that bending of the sensor did not affect its sensing performance.

### Working mechanism of photo-detectors

**Figure 8:**
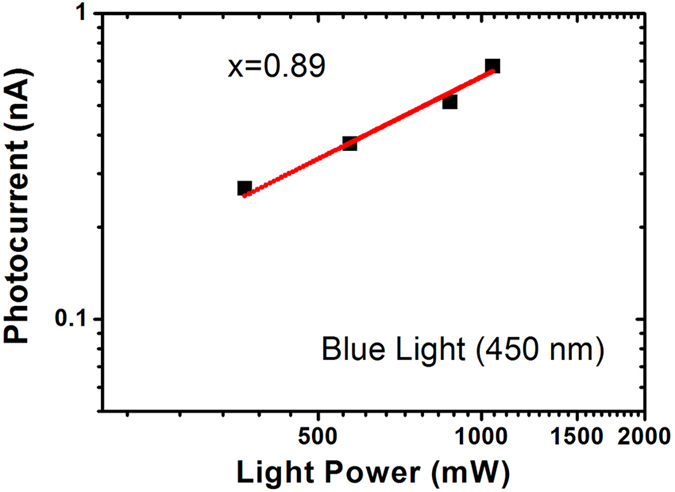
Scaling behavior of the photocurrent as a function of the blue light power at a bias voltage of 2 V.

Figure 8 shows the dependence of the photocurrent on the incident optical power extracted from Fig. 4(a). This photocurrent behavior could be fitted by a simple power law:
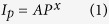
where, I_p_, A and P are the photocurrent, the proportionality constant and the power of a light source, respectively. Fitting equation (1) to the curve gives x = 0.89 for the blue light. The exponent x close to 1 indicates that the photo-electric effect is mainly contributing to the photocurrent. This result can be attributed to the high quality single crystalline CdS-NW with lower density of trap states in the energy band gap between the Fermi level and the conduction band edge. However, the value of x = 0.89 is a little lower than 1, indicating the presence of disorders in the channel layer. Considering disorders do not likely exist in the single crystalline CdS-NWs, the presence of disorder in the channel could be attributed to the charge carrier tunneling at the inter-NWs junctions.

**Figure 9:**
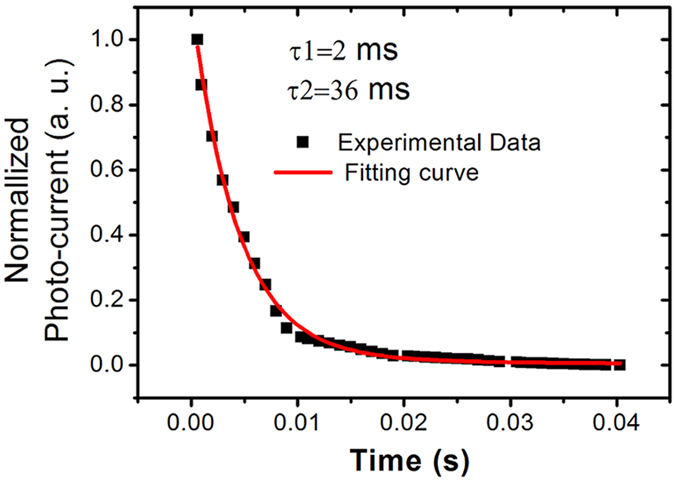
Fitting of the reset curve of the photo-detector.

The decay of photocurrent can be fitted by the bi-exponential equation (2) as reported previously^8^,^17^,^18^.
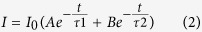
where A, B are the pre-exponential factors and τ_1_, τ_2_ are decay time constants. Typically, we assign τ_1 _< τ_2_, so that τ_1_ represents the fast component (bulk process) and τ_2_ represents the slow component (surface process). Figure 9 showsthe fitting of the experimental data (flat) using equation (2). The fast time of 2 ms is similar with the value reported in literature^17^. However, the slow delay time of 36 ms is obviously faster than reported value in literature^17^. Such, a fast response can be attributed to the three aspect of single crystalline CdS-NWs material, MCE cladding CdS-NWs structure, and excellent interconnection of NWs. The details are discussed below:

First, it was reported that the presence of charge traps as well as the local potential fluctuation in the channel defects to slow down the response time of photo-detectors^17^,^18^. For example, the defects within the NWs can cause the trapping and recombination process and generate the persistent photocurrent, which can slow down the response of CdS-NWs photo-detectors. The defects in our single crystalline CdS-NWs are fewer than polycrystalline materials. It is one of the reasons for the fast response in the work.

Second, for the photo-detectors based on the oxide semiconductor nanostructures, some researches reveal that oxygen chemisorption plays a vital role in regulating the photosensitive properties^8^,^19^. In the dark, oxygen molecules are absorbed on the surface of the oxide semiconductor as negatively charged ions by capturing free electrons in the n-type semiconductor, forming a low-conductivity depletion layer near the surface. Under light illumination, photo-excited holes migrate to the surface and discharge the absorbed oxygen ions through electron-hole recombination. The unpaired electrons thus significantly enhance the conductivity^11^. The oxygen adsorption and desorption process are usually slow, which lead to the slow response^8^,^19^. In contrast to previous photo-detectors with NWs directly exposed in air, the CdS-NWs in our devices were embedded in the MCE matrix and surrounded by cross-linked MCE polymer chains. The polymer molecules may preferentially occupy the sites where the adsorbed and ionized oxygen molecules tend to occupy. Therefore, the fast photo-response of this device can be dominated by the photon generated electrons and holes, and recombination, instead of the oxygen adsorption dominant mechanism.

Third, the interconnections of NWs dominate the charge transport in the NWs network structure. In this work, the MCE membrane treated by acetone vapor went through the re-melting and solidification process. After this process, the MCE filter membrane shrank, resulting in good interconnections between the NWs. The excellent interconnections of NWs provided short current paths and a lower contact barrier to improve the response properties remarkably.

Based on the above advantages, it is reasonable to obtain a rather fast response of our photo-detector compared to previous devices.

## Conclusions

In summary, we developed a simple but efficient method to produce high performance flexible photo-detectors based on CdS-NWs network channel and Ag-NWs network electrode. In this method, the patterns of CdS-NWs and Ag-NWs were fabricated on the MCE filter membrane using a lithographic filtration method. Then, the MCE membrane filter with device patterns was treated by alcohol and acetone vapors, and became a transparent and pore free flexible substrate. The fabricated photo-detectors exhibited UV-visible light sensitivity with response and reset time of 6 ms. In addition, the stable photoconductive characteristics of our flexible photo-detectors were maintained even the photo-detectors were bent at an angle of 90°. This research demonstrates a feasible way for the fabrication of low cost and high performance flexible photo-detectors for wearable electronics application.

## Method

### CdS-NWs synthesis and characterization

The CdS-NWs were fabricated by solvo-thermal synthesis method using L-cysteine as sulfur source as described previously^20^. The synthesis process is described as follows: 0.228 g cadmium chloride (CdCl2 2.5H2O, 1 mmol) and 0.242 g L-cysteine (C3H7NO2S, 2 mmol) were placed into a Teflon-lined stainless steel autoclave (50 mL capacity), and 40 mL ethylenediamine was added into the autoclave as a solvent. The autoclave was sealed and heated at 150 °C for 12 days, and then cooled to room temperature naturally. Yellow precipitate collected from the bottom of the autoclave was rinsed with distilled water and absolute alcohol for several times to remove the excessive reactants and byproducts. After being dried at 50 °C for 24 h, the product yellow CdS powers were collected. The morphology of CdS-NWs was characterized by JEOL scanning electron microscope (SEM). The transmission electron microscope (TEM) image, high resolution transmission electron microscopy (HRTEM) image and selected-area electron diffraction (SAED) pattern were recorded on a JEM-2010HR electron microscope. The crystalline structure of CdS-NWs was characterized by Empyrean X-ray diffraction (XRD).

### Device fabrication

CdS yellow powders were put in isopropyl alcohol (IPA) and sonicated for 1 min to prepare the NWs dispersion (typically with a concentration of 0.05 mg/mL). Commercially available Ag-NWs dispersed in IPA (2 mg/ml, Suzhou ColdStones Technology Co., Ltd.) were used as received. The lengths of the Ag NWs were 30–40 μm and diameters were within 40–100 nm.

To fabricate the CdS-NWs channel and Ag-NWs electrode, PDMS (Sylgard 184, Dow Corning) masks were fabricated by curing a degassed mixture of a base and a curer (weight ratio 10:1) at 60 °C for 2 h. For the PDMS mask of CdS-NWs channel, solidified PDMS membrane was cut with a graver to obtain the desired patterns. To fabricate the PDMS mask of Ag-NWs electrode in fork finger pattern, the degassed mixture of a base and a curer was poured into a copper stamper. After PDMS curing, the mask with the fork finger patterns was peeled off from the copper stamper.

In order to fabricate the photo-detector device, the PDMS hard masks were put on the top of the porous MCE membrane (0.45 um, 47 mm) to filter the NWs patterns. The CdS-NWs patterns were firstly filtered followed by the Ag-NWs patterns. The MCE membrane with CdS- and Ag-NWs patterns placed on an adhesive tape was sprayed by alcohol and then treated with acetone vapor for 14 min, immediately. After this process, the MCE membrane became transparent and pore-free. Finally, the MCE membrane was peeled off from the tape after it was dried at room temperature. The devices were used for testing without any further treatments.

### Photoelectrical measurements

Photoelectrical measurements were performed on a probe station equipped with a semiconductor property analyzer (Keithley 2400) at room temperature in ambient conditions. LED diodes (380 nm, 450 nm, 510 nm, and white light) were used as the illumination sources. The light power mentioned in this work was measured by integrating sphere.

## Additional Information

**How to cite this article**: Pei, Y. *et al*. CdS-Nanowires Flexible Photo-detector with Ag-Nanowires Electrode Based on Non-transfer Process. *Sci. Rep.* **6**, 21551; doi: 10.1038/srep21551 (2016).

## Figures and Tables

**Table 1:** Summary of important parameters of NWs photo-detectors.

Materials	Contact Type	Response Range	Sensitivity	Decay Time	Ref.
CdS NW	SC	<500 nm	200	240 ms	17
CdS_x_Se_1−x_ NW	SC	UV-Visible	>100	240 ms	21
Sn doped CdS NW	OC	Visible	~30	280 ms	22
β-Ga_2_O_3_ NW	OC	UV	~1000	<0.09s	23
ZnO NW	OC	UV	~180	30 s	24
ZnO-NW	OC	UV	>100	33 s	8
Bi_2_S_3_ NW	OC	Visible	24	2 ms	25
CdS NW	OC	UV-Visible	~120	6 ms	This work

Abbreviations: OC, Ohmic Contact, SC, Schottky Contact.
